# Training with an elastic bench press device provides comparable adaptations to conventional resistance training in trained men

**DOI:** 10.5114/biolsport.2026.157993

**Published:** 2026-02-06

**Authors:** Simon Gavanda, David Wischtukat, Moritz Ruckdeschel, Stephan Geisler, Steffen Held

**Affiliations:** 1 IST University of Applied Sciences, Department of Fitness & Health, Düsseldorf, Germany; 2 German Sport University Cologne, Institute for Cardiovascular Research and Sports Medicine, Cologne, Germany; 3 IST University of Applied Sciences, Department of Sport & Management, Düsseldorf, Germany

**Keywords:** Muscle mass, Powerlifting, Slingshot, Strength

## Abstract

This study investigated the effects of a 10-week powerlifting-style bench press resistance training program, with (EBD) and without an elastic bench press device (RAW), on one-repetition maximum (1-RM), body mass (BM), muscle mass (MM), fat-free mass (FFM), and muscle thickness of the triceps brachii and pectoralis major. Twenty-two trained males (EBD: n = 10, age 25.1 ± 4.5 years, relative 1-RM 1.05 ± 0.31; RAW: n = 12, age 24.9 ± 3.9 years, relative 1-RM 1.07 ± 0.31) performed training three times weekly, with the EBD group training at 10% higher intensity than the RAW group. Analysis revealed significant time effects for 1-RM (EBD: +8.5 ± 2 5.3%, d = 0.34, p ≤ 0.001; RAW: +7.8 ± 28%, d = 0.28, p ≤ 0.001) and pectoralis major muscle thickness (EBD: +15.5 ± 23.9%, d = 0.65, p = 0.007; RAW: +17.2 ± 13.2%, d = 1.31, p = 0.001). Despite significant time effects, BM revealed no relevant post hoc changes (EBD: +1.0 ± 23.8%, d = 0.04, p = 0.406; RAW: 0.0 ± 19.5%, d = 0.00, p = 1.000). No significant interaction effects emerged for any outcome, and triceps thickness, MM, FFM, and fat mass did not show statistically significant changes. EBD and RAW training produced comparable strength and hypertrophic adaptations. Although the RAW group showed slightly greater gains in pectoralis major thickness, the difference was not statistically significant. This study highlights that while EBD provides a viable alternative to conventional resistance training, it does not confer superior gains.

## INTRODUCTION

Resistance training is widely recognized for enhancing muscular strength, hypertrophy, and overall functional performance [1]. Among various strength exercises, the bench press is a fundamental compound movement, targeting key upper body muscles, including the pectoralis major, triceps brachii, and anterior deltoid [2]. The bench press is widely used for performance enhancement and is a critical component in sports such as powerlifting [3], rugby [4], American football [5] and combat sports [6]. To optimize strength adaptations, various resistance training methods, such as accommodating resistance with chains, elastic bands, or supportive devices have been employed [7, 8]. Among these, elastic bench press devices (EBDs), such as the Sling Shot^®^, have gained popularity for their claimed ability to increase training intensity and reduce joint stress [9]. EBDs are designed to support the lifter during the eccentric phase by storing elastic energy, which is released during the concentric phase, potentially enabling greater overload and strength adaptations [10].

Prior research on EBDs has produced mixed findings. Gavanda et al. [10] found that EBD resistance training, conducted over eight-week period with two sessions per week, did not yield superior strength gains compared to conventional (RAW) training despite higher training loads. Similarly, Dugdale et al. [11] and Niblock and Steele [12] observed in acute studies that, although the use of EBDs allows greater loads, it does not translate into greater neuromuscular activity or hypertrophic adaptations. Conversely, Ye et al. [9] suggested that EBDs could improve bench press mechanics and reduce joint stress. Despite these insights, there remains limited evidence regarding the chronic adaptations in muscle thickness and body composition. Furthermore, most studies have not assessed the effects of EBD resistance training on trained individuals under a structured, progressive powerlifting-style training program. This gap in knowledge highlights the need for further research to determine the long-term impact of EBDs on strength and hypertrophic outcomes.

To address this gap, the aim of our study was to compare the effects of a 10-week powerlifting-style bench press training program performed with or without an EBD on strength, muscle thickness, and body composition in trained males. We hypothesize that the use of EBDs would facilitate greater strength gains than previously reported, due to the higher training intensities applied, and may also elicit increased hypertrophic responses compared to conventional training, as our study employed a longer intervention period and a higher training frequency than that of Gavanda et al. [10]. Our findings could have significant practical implications for strength athletes and coaches, providing evidence for the efficacy of EBD for optimizing performance. Specifically, our research will provide a deeper understanding of how EBD can be incorporated into training regimens for athletes seeking to maximize both strength and muscle growth, potentially guiding future training protocols in strengthbased sports.

## MATERIALS AND METHODS

### Subjects

Based on previous research [9–12], an a priori power analysis (*α* = 0.05, study power [1-*β*-error] = 0.95, effect size partial etasquared [*η*_p_^2^] = 0.04, [*f* = 0.21], correlations among repetitive measures = 0.8; g*Power, Version 3.1.9.6) [13] revealed a required sample size of *n* = 22. To account for potential dropouts, 28 participants were initially enrolled, of whom 22 completed the study and were included in the final analysis (Table 1). All participants were men between the ages of 18 and 35, with a minimum of 1 year of experience with the bench press exercise were recruited for this study. Participants had to be healthy, with no metabolic, respiratory, or cardiovascular disorders, and no upper extremity injury in the 12 months prior to the intervention. They were required to refrain from any additional training outside of the intervention. Exclusion criteria were the use of drugs or other performance-enhancing substances, such as anabolic steroids. To be included in the final analysis, participants had to have completed at least 26 of 30 training sessions. All participants provided written informed consent and agreed to abstain from additional strength training during the intervention period. The study protocol complied with the Declaration of Helsinki and was approved by the local ethical committee (Ethic Decision No. 190718IST233). International ethical standards were met [14] and all participants signed an informed written consent after receiving all relevant study information. The participants agreed to abstain from any additional strenuous physical activity during the study. In addition, they were instructed to maintain their normal diet to minimize dietary bias, and written informed consent was obtained in advance. Furthermore, both groups did not differ (*p* ≥ 0.442; *d* ≤ 0.33) regarding height, body mass, and bench press one-repetition maximum (1-RM) (Table 1).

**TABLE 1:** Anthropometric data of both groups. Data are given as mean ± standard derivation. In addition, p value and effect size as Cohen’s d for pairwise comparison are given. RAW = bench press training without an elastic bench press device; EBD = bench press training with an elastic bench press device; 1-RM = One-repetition maximum.

Parameter	EBD	RAW	Comparison
Sample size	10 (males)	12 (males)	—

Age [yrs]	24.9 ± 3.9	25.1 ± 4.5	*p* = 0.894, *d* = 0.05

Height [m]	1.82 ± 0.07	1.80 ± 0.05	*p* = 0.442, *d* = 0.33

Body mass [kg]	94.4 ± 18.6	97.6 ± 24.0	*p* = 0.729, *d* = 0.15

1-RM [kg]	104.9 ± 28.3	106.7 ± 30.7	*p* = 0.881, *d* = 0.06

### Research Design

Following familiarization, a two-group matched-pair parallel design based on initial bench press 1-RM was used to assess the effects of a 10-week thrice-weekly, powerlifting-type upper-body resistance training program, either with (EBD) or without (RAW) an elastic bench press device, on strength, body composition, and muscle thickness in trained, healthy young adults. Familiarization, as well as all tests, were conducted in a commercial gym by the same researchers, utilizing the same equipment at the same time of day. The order of testing was anthropometric assessments (body mass [BM], fat mass [FM], fat-free mass [FFM], and muscle mass [MM]), ultrasound measurements (triceps brachii, pectoralis major), followed by 1-RM testing. Retesting was conducted 48–72 hours after the last training session.

#### Familiarization

Familiarization consisted of three training sessions, which were separated by at least 48 hours in the week prior to the intervention. The goal was to familiarize all participants with 1-RM testing and to provide technique coaching as needed. Furthermore, all participants were required to become accustomed to using an EBD (Hooke Strap Level 1, The Stronger Athlete, Alpen, Germany, sized according to the manufacturer’s recommendations based on circumference of the upper arm). To achieve this, half of the bench press training sets were completed with the EBD, and the other half were performed without it. In addition, training weights were estimated for all exercises to be implemented in week 1 of the intervention.

#### Anthropometric Testing

Anthropometric testing was performed after familiarization during the first training session before the start of the intervention and at the post-testing lab visit. BM was measured using an electronic scale (Seca 803, Seca, Hamburg, Germany) with participants wearing only underwear after they had gone to the restroom.

FFM, FM, and MM were measured using bioelectrical impedance analysis (BIA 101, Akern, Firenze, Italy) and analyzed using the BodyGram Pro software (version 3.0, Akern). To allow for fluid shift, participants laid supine for ten minutes before measurement. The skin areas where the single-use electrodes were placed were cleaned with alcohol swabs. The electrodes were applied to the right hand and foot, according to the manufacturer’s guidelines. During measurement it was ensured that participants arms and legs were not in contact with each other or any other part of the body.

The triceps brachii and pectoralis major muscle thickness on participants´ right side were measured using B-mode ultrasound imaging (DP-50, Mindray Bio-Medical Electronics Co., Ltd., Shenzhen, China) with an 8.5-MHz linear scanning head (75L53EA, Mindray Bio-Medical Electronics Co., Ltd., Shenzhen, China).

#### Performance Testing

Following five minutes of low-intensity cycling, a specific warm-up was conducted. First, all participants performed dynamic mobility exercises for the upper body, followed by four bench press warm-up sets (10 × 50%, 5 × 65%, 3 × 80%, 1 × 90% of participants´ estimated 1-RM), interspersed with 1-, 2-, 3-, and 4-minute rest periods between sets [5] on an official competition bench (IPF Competition Combo Rack, Eleiko, Halmstad, Sweden) using a training barbell (IPF Powerlifting Training Bar, Eleiko, Halmstad, Sweden) and calibrated weight plates (IPF Powerlifting Competition Plate, Eleiko, Halmstad, Sweden). Subsequently, the participants’ 1-RM was determined by gradually increasing the weight until they were unable to perform a valid repetition. The criteria for a valid repetition were based on the International Powerlifting Federation rulebook [15], with the exception of the pause on the chest. Following each attempt, participants rested for four minutes. The 1-RM was expected to be found in fewer than six trials to reduce the negative effect of fatigue. The research team provided strong verbal encouragement during the process.

#### Resistance Training Program

The supervised full-body training program lasted ten weeks, with sessions conducted three times per week, separated by 48 to 72 hours, and following a progressive undulating periodization model for the bench press exercise. In this program, repetitions were progressively reduced while intensity increased over time (see Table 2). The program was divided into two training blocks: a first block consisting of four weeks of loading, followed by one week of deloading with reduced intensity and set volume, and a second block comprising a four-week loading phase and a one-week peaking phase.

**TABLE 2:** Exercises, sets, repetition, intensity as percentage subjects’ initial one-repetition maximum, and rest periods during the intervention period.

		Week 1 & 2	Week 3 & 4	Week 5	Week 6 & 7	Week 8 & 9	Week 10
**Day 1**	Bench press	3 × 10	4 × 8	2 × 8	4 × 6	3 × 5	3 × 1
	*Bent-over barbell row*	*3 × 10*	*3 × 10*	*2 × 8*	*3 × 8*	*3 × 8*	*2 × 8*
	*Back squat*	*3 × 10*	*3 × 10*	*2 × 8*	*3 × 8*	*3 × 8*	*2 × 8*

**Day 2**	Bench press	4 × 8	4 × 6	2 × 6	4 × 4	4 × 3	Post-testing
	*Chin-ups*	*3 × 10*	*3 × 10*	*2 × 8*	*3 × 8*	*3 × 8*	
	*Deadlift*	*3 × 10*	*3 × 10*	*2 × 8*	*3 × 8*	*3 × 8*	

**Day 3**	Bench press	5 × 5	4 × 4	2 × 4	4 × 3	5 × 2	
	*Bent-over barbell row*	*3 × 10*	*3 × 10*	*2 × 8*	*3 × 8*	*3 × 8*	
	*Back squat*	*3 × 10*	*3 × 10*	*2 × 8*	*3 × 8*	*3 × 8*	

All training sessions began with a standardized warm-up identical to that used in performance testing, with the only difference being the load applied during specific warm-up sets. Participants performed the first set with ten repetitions using an empty bar, followed by a second set with ten repetitions at 50% of the load scheduled for the day. Participants trained either with an elastic bench press device (EBD) or without an EBD (RAW); in the EBD group, bench press loads were 10% higher than those in the RAW condition, accordance with previous research [10]. Cadence was standardized with a two-second eccentric phase and a concentric phase performed as fast as possible, with no rest at the top or bottom of the movement. All repetition were executed with a full range of motion, ensuring chest contact and full elbow extension.

Initial training intensity in week one was set at 65% of 1-RM for the RAW condition and 75% of 1-RM for the EBD condition. From this starting point, intensity was increased by roughly 2–3% per repetition as the prescribed repetition number decreased (week 3, 6, 8, and 10), following the NSCA’s %1-RM–repetition relationship chart [1]. Additionally, participants rated perceived exertion of all sets using the repetitions in reserve (RIR) method [16]. When RIR was rated as 0–1, the load remained unchanged; with an RIR of 2–3, the load was increased by 2.5 kg, whereas an RIR of 4–5 resulted in a 5 kg increment. Inter-set rest period was three minutes.

Subsequently, bench press training was supplemented with antagonist exercises, such as bent-over barbell rows or chin-ups, and one lower-body exercise per session (back squat or deadlift), to create a full-body training approach in a powerlifting manner without a specific strength focus. These exercises were performed within the 8–12 repetition range with two minutes rest, ensuring submaximal effort (RIR 1–2).

Participants were required to attend at least 82% of the prescribed training sessions to be included in the final analysis.

### Statistical Analysis

Data are presented as means ± standard deviation. Normality was assessed using the Shapiro–Wilk test (*p* ≥ 0.1), and homogeneity of variances was evaluated through residual plots [17]. Baseline characteristics (age, height, BM, and training experience) between the two groups (RAW vs. EBD) were compared using independent samples t-tests. For each outcome variable (bench press 1-RM, BM, FM, FFM, MM, triceps brachii, and pectoralis major thickness), a 2 (group: EBD vs. RAW) × 2 (time: pre vs. post) Aligned Rank Transform analysis of variance (ART-ANOVA) was performed [18]. The ARTANOVA method was selected because the assumptions of normality and homogeneity of variance required for a traditional ANOVA were not met. Bonferroni-corrected post hoc tests were conducted to identify significant main effects and interactions within and between groups. Effect sizes for ART-ANOVA results were reported as partial eta squared (*η*_p_^2^) and interpreted using Cohen’s thresholds [19]: small (*η*_p_^2^ = 0.01), medium (*η*_p_^2^ = 0.06), or large (*η*_p_^2^ = 0.14). Pairwise comparisons were quantified using Cohen’s *d*, interpreted as trivial (*d* < 0.2), small (0.2 ≤ *d* < 0.5), moderate (0.5 ≤ *d* < 0.8), and large (*d* ≥ 0.8) [19]. All statistical analyses were conducted using R (version 4.2.2) and RStudio (version 2023.06.0). The significance level was set at *α* = 0.05.

## RESULTS

**FIG. 1:**
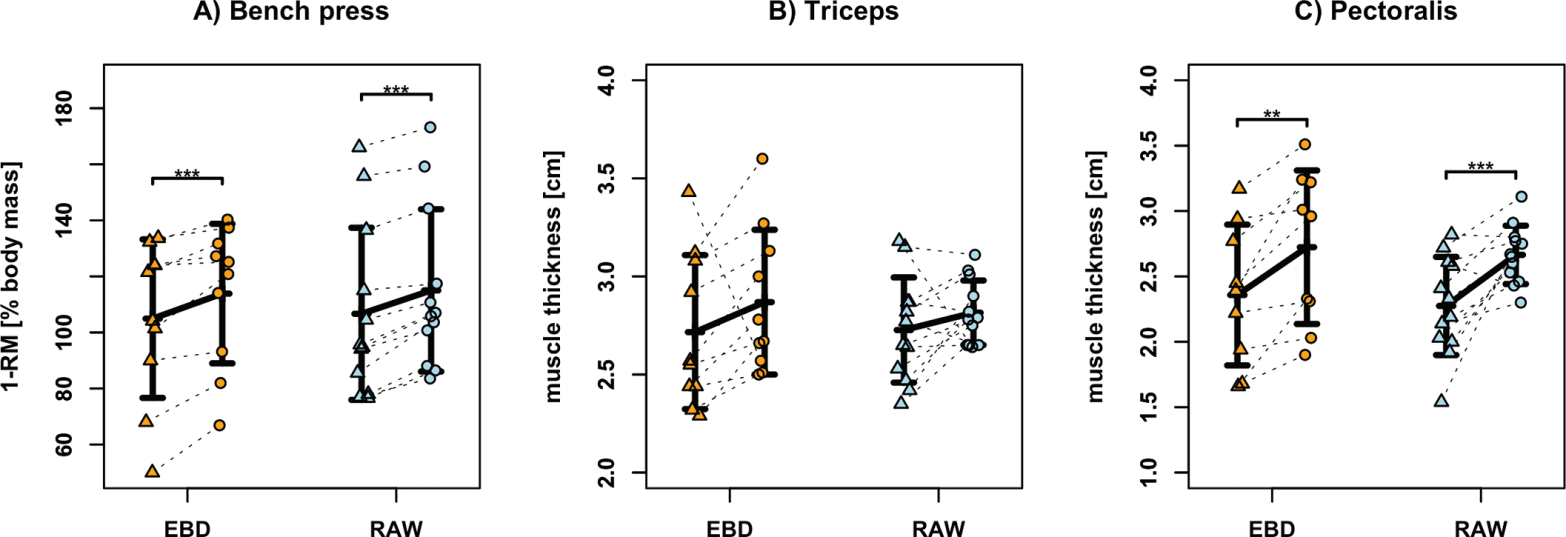
Changes in bench press one-repetition maximum (1-RM) (A), triceps brachii thickness (B), and pectoralis major thickness (C) for the training groups, either with (EBD, orange) or without (RAW, blue) an elastic bench press device. The mean changes (± standard deviation) from pre- to post-intervention, as well as the individual values, are presented. Statistical significance is indicated by asterisks: ** represents p < 0.01 and *** represents p < 0.001.

**FIG. 2:**
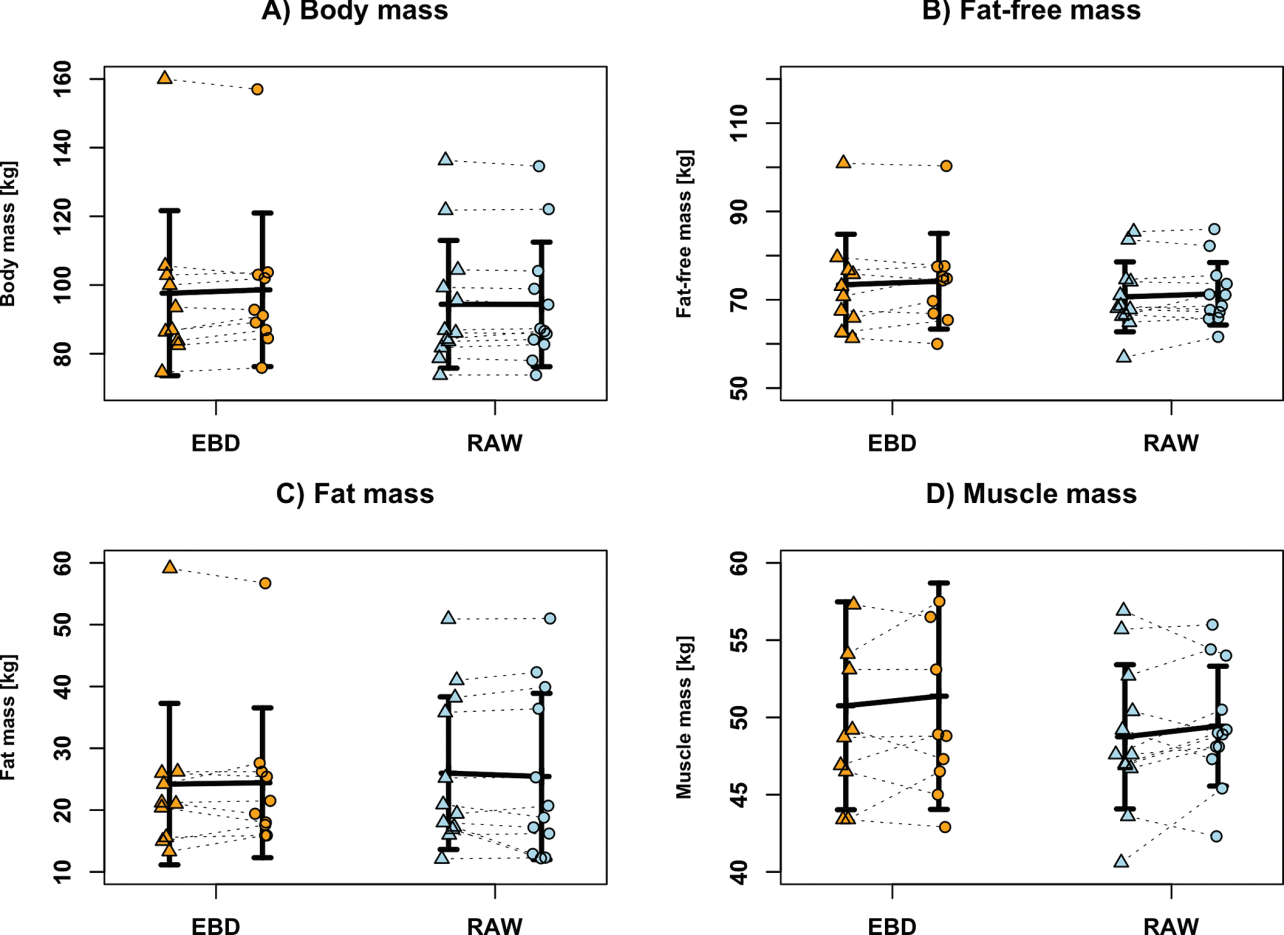
Changes in muscle mass (A), fat-free mass (B), fat mass (C), and body mass (D) for the training groups, either with (EBD, orange) or without (RAW, blue) an elastic bench press device. The mean changes (± standard deviation) from pre- to post-intervention, as well as the individual values are presented.

Adherence was 92 ± 8% in the RAW group and 95 ± 8% in the EBD group. Across all measured variables, no significant interaction effects between group (EBD vs. RAW) and time (pre vs. post) were observed (see Table 3). However, a significant time effect was found for bench press 1-RM (Figure 1A), pectoralis major thickness (Figure 1C), and BM (Figure 2D), indicating improvements from pre- to post-intervention in both groups. Specifically, post hoc testing revealed that both groups increased their bench press 1-RM, with the EBD group showing a mean improvement of 8.5 ± 25.3% (*d* = 0.34, *p* ≤ 0.001) and the RAW group 7.8 ± 28% (*d* = 0.28, *p* ≤ 0.001). Pectoralis major thickness increased significantly in both groups. Although the RAW group showed slightly greater gains (mean difference [MD] = 17.2 ± 13.2%, *d* = 1.31, *p* = 0.001) compared to the EBD group (MD = 15.5 ± 23.9%, *d* = 0.65, *p* = 0.007), this difference was not statistically significant. BM remained nearly unchanged in both the EBD (1.0 ± 23.8%, *d* = 0.04, *p* = 0.406) and the RAW (0.0 ± 19.5%, *d* = 0.00, *p* = 1.000) groups. No significant group effects were found for any variable, and all other outcomes, including FM, FFM, MM, and triceps brachii thickness showed no significant changes over time or between groups.

**TABLE 3:** Bench press one-repetition maximum (1-RM), triceps brachii muscle thickness (TB), pectoralis major muscle thickness (PM), body mass (BM), fat-free mass (FFM), fat mass (FM), and muscle mass (MM) data for the training groups, either with (EBD) or without (RAW) an elastic bench press device. The means (± standard deviation) of pre- and post-intervention data are presented for both groups. In addition, significance values (p) and effect sizes (F-values and partial eta squared [η_p_^2^]) for the interaction, group, and time effects from the Aligned Rank Transform analysis of variance (ART-ANOVA) are reported. Lastly, post hoc comparisons (mean difference [MD], Cohen´s d and significance value [p]) are provided for each relevant pairwise comparison.

Para-meter	EBD		RAW		ART-ANOVA	Post-hoc: PRE	Post-hoc: POST	Post-hoc: EBD	Post-hoc: RAW
	
	PRE	POST	PRE	POST		EBD vs. RAW	EBD vs. RAW	PRE vs. POST	PRE vs. POST
1-RM [% BM]	104.9 ± 28.3	113.9 ± 24.9	106.7 ± 30.7	115.0 ± 29.0	interaction: *F*(1,20) = 0, *ηp2* = 0.02, *p* = 0.523; group: *F*(1,20) = 0, *ηp2* = 0.00, *p* = 0.811; **time: F(1,20) = 50, *η*p2 = 0.72, p = ≤ 0.001;**	MD = -1.6 ± 27.7%, *d* = -0.06, *p* = 1.000;	MD = -1.0 ± 23.4%, *d* = -0.04, *p* = 1.000;	**MD = 8.5 ± 25.3%,** **d = 0.34,** **p = ≤ 0.001;**	**MD = 7.8 ± 28%,** **d = 0.28,** **p = ≤ 0.001;**

TB [cm]	2.72 ± 0.39	2.87 ± 0.37	2.73 ± 0.27	2.82 ± 0.16	interaction: *F*(1,20) = 0, *ηp2* = 0.00, *p* = 0.922; group: *F*(1,20) = 0, *ηp2* = 0.01, *p* = 0.723; time: *F*(1,20) = 4, *ηp2* = 0.15, *p* = 0.071;	MD = -0.4 ± 12.1%, *d* = -0.03, *p* = 1.000;	MD = 1.9 ± 9.5%, *d* = 0.20, *p* = 1.000;	MD = 5.6 ± 14%, *d* = 0.4, *p* = 0.622;	MD = 3.2 ± 7.9%, *d* = 0.41, *p* = 1.000;

PM [cm]	2.36 ± 0.54	2.72 ± 0.59	2.27 ± 0.38	2.66 ± 0.22	interaction: *F*(1,20) = 0.00, *ηp2* = 0, *p* = 0.964; group: *F*(1,20) = 0, *ηp2* = 0.01, *p* = 0.671; **time: F(1,20) = 26, *η*p2 = 0.58, p = ≤ 0.001;**	MD = 3.7 ± 20.1%, *d* = 0.18, *p* = 1.000;	MD = 2.2 ± 15.2%, *d* = 0.14, *p* = 1.000;	**MD = 15.5 ± 23.9%,** **d = 0.65,** **p = 0.007;**	**MD = 17.2 ± 13.2%,** **d = 1.31,** **p = 0.001;**

BM [kg]	97.6 ± 24.0	98.6 ± 22.4	94.4 ± 18.6	94.4 ± 18.1	interaction: *F*(1,20) = 3, *ηp2* = 0.15, *p* = 0.079; group: *F*(1,20) = 0, *ηp2* = 0.02, *p* = 0.543; **time: F(1,20) = 5, *η*p2 = 0.19, p = 0.044;**	MD = 3.4 ± 22.6%, *d* = 0.15, *p* = 1.000;	MD = 4.5 ± 21.5%, *d* = 0.21, *p* = 0.212;	MD = 1.0 ± 23.8%, *d* = 0.04, *p* = 0.406;	MD = 0.0 ± 19.5%, *d* = 0.00, *p* = 1.000;

FFM [kg]	73.4 ± 11.4	74.2 ± 10.8	70.7 ± 7.9	71.4 ± 7.1	interaction: *F*(1,20) = 1, *ηp2* = 0.04, *p* = 0.402; group: *F*(1,20) = 0, *ηp2* = 0.01, *p* = 0.615; time: *F*(1,20) = 3, *ηp2* = 0.12, *p* = 0.114;	MD = 3.9 ± 13.7%, *d* = 0.29, *p* = 1.000;	MD = 4.0 ± 12.5%, *d* = 0.32, *p* = 1.000;	MD = 1.0 ± 15.2%, *d* = 0.07, *p* = 1.000;	MD = 1.0 ± 10.6%, *d* = 0.09, *p* = 1.000;

FM [kg]	24.2 ± 13.1	24.4 ± 12.1	26.0 ± 12.3	25.4 ± 13.5	interaction: *F*(1,20) = 3, *ηp2* = 0.13, *p* = 0.097; group: *F*(1,20) = 0, *ηp2* = 0.00, *p* = 0.897; time: *F*(1,20) = 0, *ηp2* = 0.01, *p* = 0.708;	MD = -6.8 ± 48.9%, *d* = -0.14, *p* = 1.000;	MD = -4.0 ± 50.3%, *d* = -0.08, *p* = 1.000;	MD = 0.9 ± 52%, *d* = 0.02, *p* = 1.000;	MD = -2.1 ± 49.7%, *d* = -0.04, *p* = 1.000;

MM [kg]	50.8 ± 6.7	51.4 ± 7.3	48.7 ± 4.7	49.4 ± 3.9	interaction: *F*(1,20) = 0.00, *ηp2* = 0.02, *p* = 0.510; group: *F*(1,20) = 0, *ηp2* = 0.01, *p* = 0.753; time: *F*(1,20) = 2, *ηp2* = 0.10, *p* = 0.157;	MD = 4.1 ± 11.7%, *d* = 0.35, *p* = 1.000;	MD = 3.9 ± 11.3%, *d* = 0.35, *p* = 1.000;	MD = 1.2 ± 13.8%, *d* = 0.09, *p* = 1.000;	MD = 1.4 ± 8.8%, *d* = 0.16, *p* = 1.000;

## DISCUSSION

Our study investigated the effects of a 10-week powerlifting-style bench press resistance training program, with and without an elastic bench press device, on strength, muscle mass, and body composition in trained males. Both groups demonstrated relevant improvements over time in bench press 1-RM, pectoralis major thickness, and body mass. However, no significant differences were observed between groups for any measured outcomes. Despite the EBD group’s higher as well as the individual values are presented. training intensity, hypertrophic and strength adaptations were comparable to those achieved through conventional (RAW) training.

Our findings align with previous research indicating no significant advantage of using an EBD over conventional bench press training for strength and hypertrophic adaptations [10]. Similar to Dugdale et al. [11] and Niblock & Steele [12], we observed that although the EBD group trained with higher loads, the resulting increases in 1-RM and muscle thickness were comparable to those in the RAW group. This supports the theory that the elastic support during the eccentric phase limits true overload, thereby reducing neuromuscular stimulus during the lift [9]. Specifically, the EBD contributes external work by storing and releasing elastic energy, particularly in the bottom position [10]. Consequently, part of the load is assisted by the EBD rather than generated solely by the trainee, which may limit maximal neuromuscular activation and prevent the full recruitment of motor units that would otherwise occur under traditional resistance conditions. Nevertheless, this partial overload at the end of the lift does not appear to provide additional advantages for improving 1-RM strength in trained individuals.

Interestingly, our findings contrast with those of Lasevicius et al. [21], who reported greater triceps hypertrophy after 10 weeks of resistance training, whereas no such increase was observed in our study. This discrepancy may be explained by differences in training design, as they employed a hypertrophy-oriented program (8–12 repetitions) that included, in addition to 12 weekly sets of the bench press, another 12 weekly sets of triceps pushdown [20]. In contrast, our program did not include triceps-specific exercises, resulting in a lower overall triceps training volume. Additionally, our use of ultrasound for muscle thickness measurement may have provided more precise insights compared to circumference measurements used in Gavanda et al. [10].

Despite higher training intensities in the EBD group, no significant differences in BM or MM changes emerged. This echoes findings from Niblock & Steele [12], who highlighted that EBD’s increased load potential may primarily enhance mechanical advantage rather than stimulate additional hypertrophy. Collectively, these results emphasize that EBDs may have limited value for enhancing chronic strength or muscle growth in trained individuals but could still serve as a tool for training variation or injury management.

Despite the relevance of our findings, several limitations should be acknowledged. First, the relatively small sample size may have limited statistical power, potentially masking subtle group differences. Additionally, the 10-week intervention period, though consistent with similar research, may have been too short to detect long-term hypertrophic adaptations. Our use of BIA for body composition, while practical, is less precise than gold-standard methods such as dual energy x-ray absorptiometry (DEXA). However, this study offers notable strengths. The randomized controlled design and rigorous familiarization procedures enhance the reliability of our results. Additionally, using ultrasound for muscle thickness measurements provides more precise insights than circumference-based assessments used in prior research [10]. Our comparison of EBD and RAW training within a structured, powerlifting-style program offers valuable insights into their relative efficacy, addressing an important gap in the literature. Future research should employ longer intervention periods and focus on other populations, such as youth athletes or female participants, to better understand the chronic effects of EBD training. Additionally, studies could further focus on upper-body performance measures, such as speed, power, or technical aspects, investigating potential changes in bar path following EBD training.

## CONCLUSIONS

In conclusion, our study demonstrates that an EBD bench press training program results in comparable strength and hypertrophic outcomes to traditional resistance training in trained males. Despite the higher training intensities facilitated by EBD, no significant group differences were observed. These findings suggest that EBD may serve as a useful variation tool for experienced lifters but is unlikely to provide superior strength or hypertrophy gains compared to conventional methods. Coaches and practitioners can incorporate EBD as part of a varied training regimen, but it should not be relied upon as a primary method for maximizing strength adaptations.
